# Serial FDG‐PET in probable seronegative autoimmune encephalitis presenting as severe behavioral regression in a child with autism

**DOI:** 10.1002/pcn5.70395

**Published:** 2026-09-07

**Authors:** Edouard Guez, Theodore Falempin, Cyril Hanin, Alison Arbouche, Rony Boucher, Myriam Hamdi, Marie Raffin, Angèle Consoli, Aurélie Kas, Marianna Giannitelli, David Cohen

**Affiliations:** ^1^ Department of Child and Adolescent Psychiatry Pitié‐Salpêtrière Hospital, AP‐HP Sorbonne University Paris France; ^2^ INSERM UMR‐S1136, Institute Pierre Louis Epidemiology and Public Health (iPLesp), Team Social Epidemiology, Mental Health and Addictions (ESSMA) Sorbonne University Paris France; ^3^ Department of Child and Adolescent Psychiatry Centre de Référence des Maladies Rares à Expression Psychiatrique & PSYDEV Team, Pitié‐Salpêtrière University Hospital, AP‐HP Sorbonne University Paris France; ^4^ AP‐HP, Hôpitaux Universitaires Pitié‐Salpêtrière Charles Foix, Service de Médecine Nucléaire and LIB, INSERM U1146 Sorbonne Université Paris France; ^5^ Institute for Intelligent Systems and Robotics, CNRS UMR 7222 Sorbonne University Paris France

**Keywords:** autism, autoimmune encephalitis, behavioral regression, case report, seronegative

## Abstract

**Background:**

Autoimmune encephalitis in children may present with predominant psychiatric and behavioral symptoms, creating diagnostic challenges, particularly in patients with pre‐existing autism spectrum disorder. Seronegative forms are especially difficult to recognize because neuronal autoantibodies are not detected, and diagnostic overshadowing may delay consideration of immune‐mediated etiologies.

**Case Presentation:**

We report the case of an 11‐year‐old boy of Turkish background living in France, with autism and normal intelligence, who developed severe subacute behavioral and cognitive regression. Symptoms included explosive aggression, self‐injurious behavior, obsessive‐compulsive‐like behavioral loops, psychotic‐like features, agitated catatonic deterioration from baseline, loss of adaptive skills, and marked functional decline. The condition was resistant to intensive psychiatric care and multiple psychotropic treatments. Etiological assessment revealed focal epilepsy, cerebrospinal fluid abnormalities, and abnormal brain fluorodeoxyglucose positron emission tomography (FDG‐PET) findings, while extensive work‐up excluded infectious, metabolic, genetic, systemic autoimmune, and oncological causes. Neuronal autoantibodies were negative in serum and cerebrospinal fluid. A cautious diagnosis of probable seronegative autoimmune encephalitis was retained based on the clinical course, focal epilepsy, serial FDG‐PET abnormalities, follow‐up cerebrospinal fluid findings, exclusion of alternative etiologies, and expert consensus. Intravenous immunoglobulin therapy was associated with progressive but partial clinical improvement, and rituximab was introduced after a response plateau.

**Conclusion:**

This case highlights the risk of diagnostic overshadowing in children with autism presenting with severe, treatment‐resistant behavioral or catatonic regression. Immune‐mediated etiologies should be considered even in the absence of detectable autoantibodies. Serial FDG‐PET may support diagnostic reasoning and follow‐up, while remaining nonspecific and insufficient as diagnostic proof in isolation.

## BACKGROUND

Children with autism may develop acute or subacute behavioral deterioration, catatonic deterioration from baseline, or severe externalizing symptoms that risk being attributed solely to autism.^1^ Autism involves persistent social‐communication difficulties and restricted/repetitive behaviors, including stereotyped, self‐injurious, compulsive, ritualistic, and sameness behaviors, which may be linked to emotional regulation and adaptive functioning.^2^ Because severe behavioral presentations in autism spectrum disorder (ASD) are etiologically nonspecific, comprehensive assessment is required to identify co‐occurring medical or neurological contributors and avoid diagnostic overshadowing of treatable organic or immune‐mediated etiologies.^3^, ^4^

Autoimmune encephalitis (AE) may present in children with predominant psychiatric or behavioral symptoms, including agitation, aggression, psychotic features, cognitive dysfunction, and functional decline.^5^ Seronegative AE is especially challenging because neuronal autoantibodies are absent, and diagnosis relies on the overall syndrome, cerebrospinal fluid (CSF) and neuroimaging findings, exclusion of alternatives, and cautious interpretation of treatment response.^6^ Delayed recognition may prolong psychiatric management and postpone immunomodulatory therapy, particularly when neuropsychiatric symptoms predominate.^7^, ^8^ ASD and AE rarely coexist, but regression may be misread as autistic progression or psychiatric comorbidity.^9^, ^10^ We report an 11‐year‐old boy with ASD and normal intelligence who developed severe behavioral regression with catatonia‐like deterioration, prompting multidisciplinary assessment for a possible immune‐mediated etiology.

## CASE PRESENTATION

### Patient history

The patient was an 11‐year‐old boy of Turkish family background living in France with both parents and a younger sibling. He had a prior clinical diagnosis of ASD based on DSM‐5 criteria; no baseline Autism Diagnostic Observation Schedule (ADOS) or Autism Diagnostic Interview‐Revised (ADI‐R) assessment was identified, and no intellectual disability was documented. Before the episode, he was verbally communicative, continent, attended mainstream school with support, and had preserved cognitive abilities. Developmental history included delayed language acquisition, preserved gross motor development, benign febrile seizures in infancy, and no known later neurological sequelae. During the early regression, he developed Epstein–Barr virus (EBV)/parvovirus B19‐associated hepatitis, which resolved spontaneously with liver normalization within approximately 15 days. Over several months, he showed marked decline from baseline, with loss of adaptive skills, daily enuresis and encopresis, refusal to wear clothes, disorganized eating behaviors such as eating from the floor, pronounced social withdrawal, and severe impairment in daily living, refractory to standard psychiatric management. Family history included obsessive‐compulsive and anxiety/depressive symptoms, but no known autoimmune or neuroinflammatory disorder.

### Clinical findings

He was admitted to our tertiary child and adolescent psychiatry department for severe behavioral disturbance and subacute cognitive regression. At admission, he was afebrile and hemodynamically stable, with preserved general condition and no dysmorphic, cutaneous, cardiopulmonary, or abdominal abnormality. Neurological examination was limited by poor cooperation but showed preserved consciousness and orientation, no cranial nerve deficit, generalized hypotonia, and impaired motor coordination. Recurrent abnormal motor phenomena were reported or observed, including dystonic‐like jaw and tongue movements, intermittent right‐sided motor manifestations, and ocular deviation, raising concern for focal seizures, paroxysmal neurological events, or medication‐related extrapyramidal phenomena.

The clinical picture included frequent aggression toward others, self‐injury, intense anxiety, emotional dysregulation, explosive outbursts, severe obsessive‐compulsive‐like “behavioral loops,” echolalia, verbal perseverations, marked intolerance to transitions, social withdrawal, and severe behavioral crises requiring protective measures. Psychotic‐like phenomena included soliloquies, fixed staring, fearful behaviors, and apparent responses to internal stimuli. Overall, the presentation suggested catatonic deterioration from baseline rather than primary catatonia, with psychomotor and behavioral disorganization, negativism‐like responses, stereotyped verbal and motor phenomena, withdrawal, self‐injury, and profound loss of adaptive functioning. These manifestations overlapped with severe ASD‐related dysregulation and encephalitis‐related motor phenomena, requiring cautious interpretation.

### Timeline

**Figure 1:**
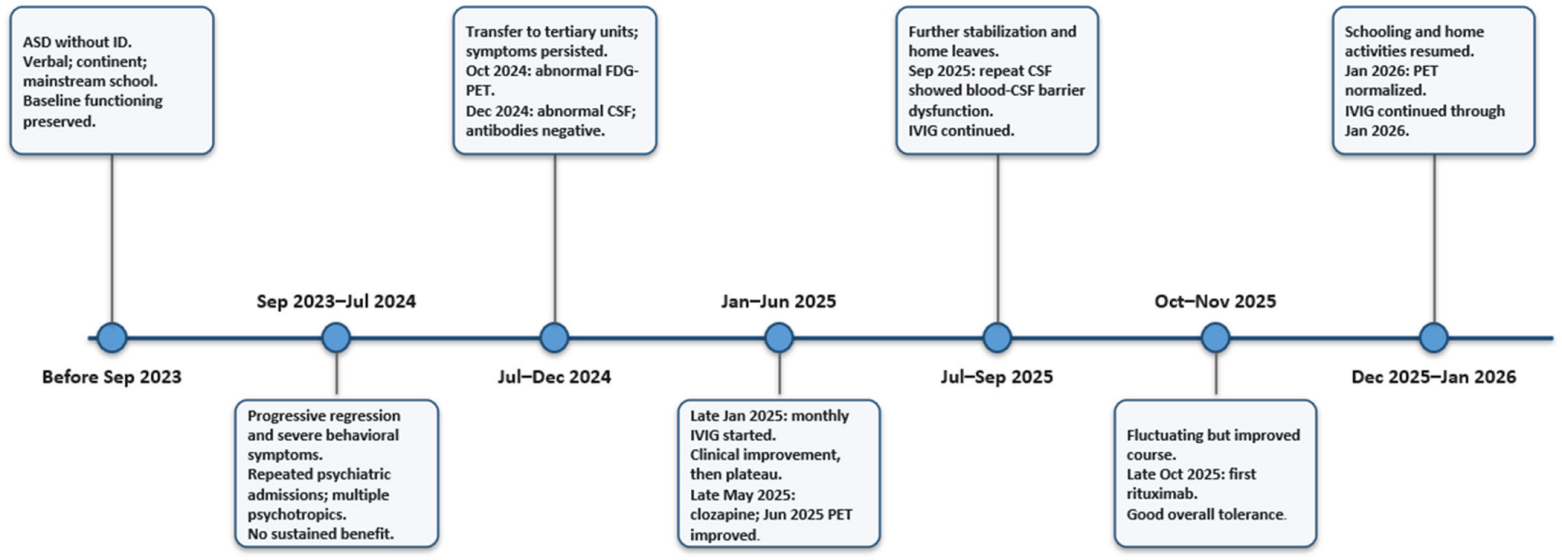
Clinical timeline. The timeline summarizes the main clinical, diagnostic, therapeutic, and functional milestones. Dates are approximate when events occurred progressively over several weeks or months. ASD, autism spectrum disorder; CSF, cerebrospinal fluid; FDG‐PET, fluorodeoxyglucose positron emission tomography; IVIG, intravenous immunoglobulins.

The main milestones are summarized in Figure 1. Supporting Information S1: Table S1 provides the detailed treatment chronology with doses and behavioral, electroencephalography (EEG), and fluorodeoxyglucose positron emission tomography (FDG‐PET) evolution.

### Etiological assessment

Given the atypical subacute course, severe functional regression, and poor response to standard psychiatric treatment, an extensive diagnostic work‐up was undertaken. The detailed ASD, infectious, neuronal antibody, autoimmune, thyroid, metabolic/genetic, and oncological work‐up is summarized in Supporting Information S2: Table S2. Brain magnetic resonance imaging (MRI) showed no significant structural abnormality apart from a small incidental right frontal arachnoid cyst. Initial EEG showed bitemporal slow irregularities and occasional right frontocentral epileptiform sharp‐wave bursts, supporting focal epilepsy; subsequent EEGs no longer showed epileptiform abnormalities after antiepileptic treatment.

**Figure 2:**
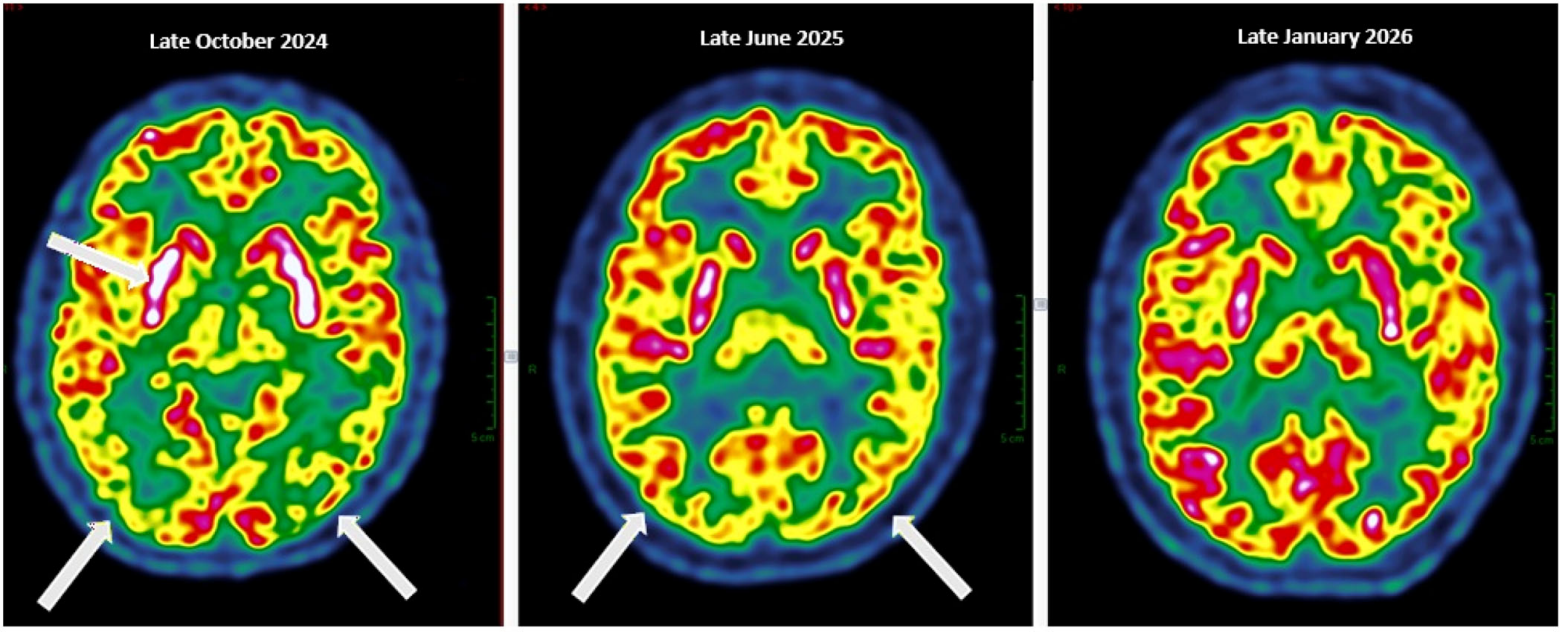
Brain fluorodeoxyglucose positron emission tomography (FDG‐PET) before and after immunomodulatory treatment. Representative axial FDG‐PET images are shown for late October 2024, late June 2025, and late January 2026. FDG‐PET examinations were performed after clinical premedication administered by the care team according to the unit's usual strategy for behaviorally constrained examinations, adapted to the patient's current treatment regimen. The initial FDG‐PET showed bilateral posterior associative cortical hypometabolism, predominantly occipital, associated with bilateral symmetric striatal hypermetabolism, without procedural sedation or documented motion artifact in the nuclear medicine report. Follow‐up FDG‐PET in late June 2025 showed marked improvement of these abnormalities. The final FDG‐PET in late January 2026 was reported as being within normal limits overall, with normalization of striatal metabolism and disappearance of the posterior associative cortical abnormalities, despite motion artifacts, reduced acquisition duration, and mild nonspecific bilateral mediofrontal hypometabolism.

Brain FDG‐PET performed in late October 2024 revealed bilateral posterior associative cortical hypometabolism, predominantly occipital, with bilateral symmetric striatal hypermetabolism. Because the patient was behaviorally constrained, the examination was performed after clinical premedication; for the initial FDG‐PET, documented premedication included amisulpride 400 mg, diazepam 20 mg, melatonin 12 mg, clonidine 0.15 mg, and intramuscular hydroxyzine 50 mg. No procedural sedation or motion artifact was documented in the nuclear medicine report for the initial examination. Serial follow‐up FDG‐PET showed progressive improvement and later near‐normalization of these abnormalities (Figure 2).

The first CSF analysis in December 2024 was limited by a traumatic hemorrhagic lumbar puncture, making CSF biochemistry and intrathecal synthesis assessment insufficient for inflammatory diagnosis; direct examination, infectious testing, cytokines, interferon‐alpha, and neuronal antibody testing were negative. Repeat CSF analysis in September 2025 showed mild pleocytosis, elevated CSF albumin compatible with blood–CSF barrier dysfunction, and no clear intrathecal immunoglobulin G (IgG) synthesis; neuronal autoantibodies remained negative in serum and CSF.

Infectious, systemic autoimmune, thyroid‐related, oncological, paraneoplastic, metabolic, and genetic investigations did not identify an alternative diagnosis. In particular, CSF herpes simplex virus (HSV)1/2 and EBV polymerase chain reaction (PCR), multiplex meningitis/encephalitis panel, and Lyme testing were negative; prior EBV/parvovirus B19 hepatitis was interpreted as a resolved systemic infection rather than active viral encephalitis. Low‐titer antinuclear antibodies were present, but anti‐double‐stranded DNA antibodies (anti‐dsDNA), antineutrophil cytoplasmic antibodies (ANCA), and thyroid antibodies were negative, without evidence for neuropsychiatric systemic lupus erythematosus or Hashimoto encephalopathy. Whole‐body computed tomography (CT) and targeted ultrasound did not identify a tumor, despite mild alpha‐fetoprotein (AFP) elevation requiring monitoring.

Overall, based on subacute neuropsychiatric deterioration, focal epilepsy, dynamic FDG‐PET abnormalities, follow‐up CSF findings, exclusion of alternative etiologies, and multidisciplinary consensus, a cautious diagnosis of probable seronegative AE was retained.

### Therapeutic intervention

During hospitalization, the patient received combined psychiatric, behavioral, neurological, and immunomodulatory interventions. Before an immune‐mediated etiology was considered, multiple psychotropic strategies and structured behavioral measures had not produced sustained global improvement. After focal epileptiform abnormalities and paroxysmal motor manifestations were identified, antiepileptic treatment was optimized with valproate up to 800 mg/day and lacosamide 200 mg/day; subsequent EEGs no longer showed epileptiform abnormalities. The detailed chronology is provided in Supporting Information S1: Table S1.

Once probable seronegative AE was considered, monthly intravenous immunoglobulins (IVIG) was initiated (Table 1). The first course was administered over four days at 30 g/day, and subsequent courses generally over three days using a 30–40–50 g schedule. Protective measures were occasionally required during infusions to prevent self‐injury or treatment interruption. After initial improvement followed by fluctuations and plateau, rituximab 750 mg/m^2^, approximately 1 g, was introduced and well tolerated.

**Table 1:** Immunomodulatory treatment protocol.

Treatment	Step	Dose/preparation	Timing/schedule	Tolerance/comments
IVIG	First course	Privigen 30 g/day for 4 days (total: 120 g)	Mid‐Jan 2025	Polaramine premedication; vital‐sign and skin monitoring. No infusion‐related adverse event reported.
IVIG	Monthly maintenance courses	Usually privigen 30–40–50 g over 3 days (total: 120 g/cycle)	Monthly courses from Feb 2025; continued through Jan 2026	Good overall tolerance. Concomitant hydration used during subsequent courses to shorten mechanical‐restraint time during infusions.
IVIG	Observed clinical course	–	After repeated courses	Approx. 40% clinical improvement after first three courses; symptom recrudescence before next course; later partial response/plateau.
IVIG	Delayed final course	Usually Privigen 30–40–50 g over 3 days (total: 120 g/cycle)	Mid‐Jan 2026 (delayed after flu‐like illness)	No adverse event reported; delay associated with increased loops, irritability, and frustration intolerance.
Rituximab	Pre‐infusion data	Weight 67.5 kg; body surface area 1.7562 m^2^	First infusion: end of Oct 2025	Second‐line immunotherapy after partial response and plateau under IVIG. Close allergy surveillance recommended.
Rituximab	Hydration and premedication	NaCl 0.9% 1 L; dexchlorpheniramine 5 mg IV; paracetamol 950 mg IV; methylprednisolone 65 mg IV	Hydration from 08:00; premedication around 08:30	Premedication included corticosteroids according to protocol, and was administered 30 min before rituximab to reduce infusion‐related reactions.
Rituximab	Infusion dose	750 mg/m^2^, capped at 1 g total; in this patient: 1 g diluted in 500 mL NaCl 0.9%	Start around 09:00	Good neurological and hemodynamic tolerance; no severe infusion‐related adverse event reported.
Rituximab	Infusion‐rate escalation	25, 50, 100, 150, 200, 250, 300, 350 mg/h for 30 min each, then 400 mg/h for 43 min	Escalation every 30 min; total duration approx. 4 h 45 min to 5 h	Enhanced supervision and protective measures required because of behavioral agitation.
Rituximab	Monitoring/safety	HR/RR/BP/SpO_2_/temperature/skin status; emergency equipment available	Every 15 min during first hour, then every 30 min; 2 h post‐infusion observation	No severe infection, hematological complication, or neurological worsening reported during follow‐up.

*Note*: IVIG was used as first‐line immunotherapy, followed by rituximab as second‐line therapy after partial response and clinical plateau. Clinical response was considered supportive but not diagnostic in isolation.

Abbreviations: BP, blood pressure; HR, heart rate; IV, intravenous; IVIG, intravenous immunoglobulins; RR, respiratory rate; SpO_2_, peripheral oxygen saturation.

In parallel, psychiatric medications were adjusted. Before immunotherapy, antipsychotic, antidepressant, benzodiazepine, and mood‐stabilizing/antiepileptic strategies had not produced sustained improvement. Clozapine was introduced in late May 2025 for persistent psychotic‐like symptoms and severe behavioral dysregulation and increased to 350 mg/day by late September 2025. Improvement coincided with repeated IVIG, clozapine titration, antiepileptic treatment, and structured psychosocial/educational rehabilitation.

### Follow‐up and outcomes

**Figure 3:**
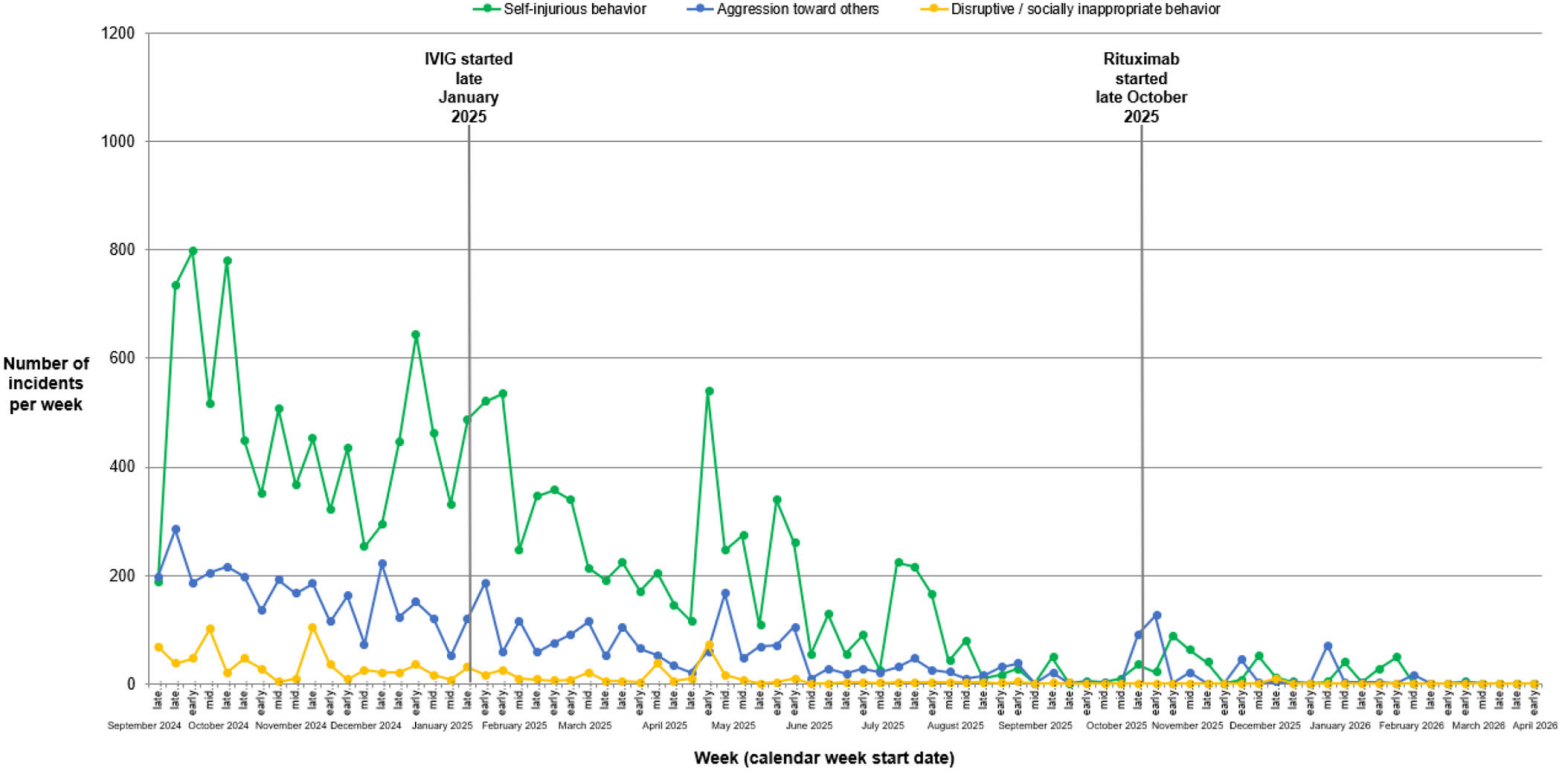
Evolution of the behavioral profile of the patient from September 2024 to April 2026. Curves represent the weekly number of recorded incidents in three behavioral domains: self‐injurious behavior, aggression toward others, and disruptive/socially inappropriate behavior. Incidents were recorded during full‐time hospitalization until January 2026 and subsequently during twice‐weekly day‐hospital follow‐up at the same specialized unit. Vertical reference lines indicate the start of intravenous immunoglobulins (IVIG) in late January 2025 and rituximab in late October 2025.

Follow‐up showed progressive but incomplete improvement, without immediate full response after the first IVIG course. After repeated courses, aggression, self‐injury, psychomotor agitation, behavioral disorganization, and psychotic‐like symptoms gradually decreased, although fluctuations persisted around frustration, transitions, hygiene, treatment administration, and periods before subsequent IVIG courses. Weekly behavioral monitoring data are shown in Figure 3.

In parallel, the patient became more emotionally available and socially engaged, partially recovered adaptive skills, and tolerated family visits, home leaves, activities outside hospital, and short hospital‐based schooling. Full‐time hospitalization was discontinued, followed by twice‐weekly day‐hospital care. Follow‐up EEGs no longer showed epileptiform abnormalities, serial FDG‐PET paralleled clinical improvement (Figure 2), and no severe infusion‐related reaction, severe infection, hematological complication, or neurological worsening was observed.

## DISCUSSION

Pediatric AE, including seronegative forms, may present with prominent neuropsychiatric symptoms such as psychosis, behavioral disturbance, catatonia, autistic regression, cognitive decline, and functional deterioration.^11^ Neuronal autoantibodies may remain negative despite supportive neuroimaging or CSF findings.^12^, ^13^ AE may also produce autism‐like or broader neurodevelopmental presentations in selected regressive or atypical presentations of ASD,^9^ and suspected AE is particularly difficult to recognize in children with baseline neurodevelopmental disorders because of symptom overlap and risk of delayed diagnosis.^10^

AE remains a clinical diagnosis supported by paraclinical investigations and exclusion of alternative etiologies. Strictly applying Graus et al. criteria, the present case fulfilled criteria for possible AE, not definite AE,^14^ because MRI was non‐contributory, neuronal autoantibodies were negative, and the first hemorrhagic CSF sample was poorly interpretable. The term probable seronegative AE was therefore used cautiously after multidisciplinary assessment, integrating rupture from premorbid functioning, focal epilepsy, dynamic FDG‐PET abnormalities, follow‐up CSF findings, exclusion of major infectious, metabolic, systemic autoimmune, genetic, and oncological alternatives, and longitudinal clinical/paraclinical evolution. This approach is consistent with pediatric recommendations emphasizing multimodal assessment without relying on antibody positivity alone,^7^ and with expert discussions stressing strict criteria, exclusion of mimics, and robust serum/CSF antibody testing in antibody‐negative cases.^15^

Pathophysiologically, this case suggests a possible antibody‐negative immune‐mediated neuropsychiatric syndrome rather than classic antibody‐defined AE. Repeat CSF showed elevated albumin compatible with blood–CSF barrier dysfunction, without clear intrathecal IgG synthesis. Prior EBV/parvovirus B19‐associated hepatitis may have contributed to immune activation, but this remains speculative because the hepatitis resolved systemically, CSF EBV PCR was negative, and no pathogen‐specific CNS infection was identified. While this single case cannot serve as definitive proof, it provides strong clinical support for the hypothesis of non‐antibody‐specific autoimmune psychosis and highlights the need to explore multi‐stage post‐infectious mechanisms in future studies.

Catatonia‐like features also required cautious interpretation. In neurodevelopmental disorders, catatonia may overlap with stereotypies, obsessive‐compulsive‐like symptoms, language abnormalities, motor phenomena, anxiety‐driven opposition, and severe behavioral dysregulation. Hauptman et al.^1^ proposed catatonic deterioration from baseline to identify catatonic features exceeding the premorbid neurobehavioral profile. In this patient, rupture from baseline, psychomotor and behavioral disorganization, echolalia, stereotyped verbal and motor phenomena, negativism‐like responses, withdrawal, self‐injury, and profound functional regression supported this interpretation, whereas absence of mutism, stupor, posturing, or clear response to zolpidem or benzodiazepines weakened support for classic primary catatonia.

Differential diagnoses included pediatric acute‐onset neuropsychiatric syndrome (PANS)/pediatric autoimmune neuropsychiatric disorders associated with streptococcal infections (PANDAS), primary psychiatric or obsessive‐compulsive disorder, medication‐induced toxicity, epileptic encephalopathy, metabolic or genetic disorders, infectious encephalitis, and paraneoplastic or oncological conditions. PANS/PANDAS were considered because they may include obsessive‐compulsive symptoms or tics, emotional lability, irritability or aggression, regression, sensory or motor abnormalities, sleep disturbances, and urinary symptoms,^16^, ^17^ but were less likely given the progressive onset, absence of documented streptococcal trigger, focal epilepsy, abnormal FDG‐PET findings, CSF abnormalities, and broader evidence of CNS involvement. The rupture from premorbid functioning, psychotic‐like manifestations, abnormal motor phenomena, catatonia‐like features, and resistance to multiple psychotropic strategies also argued against isolated psychiatric disorder or simple exacerbation of autism‐related features, illustrating the risk of diagnostic overshadowing in ASD.^18^

Brain FDG‐PET was supportive because MRI was non‐contributory and clinical assessment was complicated by ASD, behavioral fluctuations, rehabilitation, antiepileptic treatment, and clozapine. A previous report from our group described probable seronegative AE with catatonia in a 16‐year‐old without premorbid neurodevelopmental disorder, in which serial FDG‐PET supported diagnosis, therapeutic decision‐making, and monitoring.^19^ The present case showed a partly overlapping longitudinal pattern, with posterior associative cortical hypometabolism and bilateral striatal hypermetabolism followed by marked improvement and later near‐normalization, but differed by younger age, pre‐existing ASD, and the central role of diagnostic overshadowing. Indirect support also comes from systemic autoimmune disease, as Saito et al.^20^ reported regional cerebral hypometabolism in systemic lupus erythematosus with major depressive disorder. Together, these observations support the relevance of functional imaging in selected immune‐mediated neuropsychiatric states, while emphasizing that metabolic patterns are nonspecific and insufficient as diagnostic proof.

The clinical evolution under immunomodulatory treatment was compatible with, but did not prove, an immune‐mediated process. Pediatric AE recommendations support early immunotherapy when clinical and paraclinical findings are consistent with AE,^7^ and rituximab was introduced after partial response and plateau under first‐line treatment. However, pediatric antibody‐negative cases are heterogeneous and may show limited or unsustained responses when consensus criteria are not fully met.^21^ Because this was a single case involving multimodal care, the respective contributions of IVIG, rituximab, clozapine, antiepileptic treatment, and structured rehabilitation cannot be determined. Therapeutic response was therefore interpreted as supportive only when integrated with the broader clinical, CSF, EEG, and serial FDG‐PET evolution.

FDG‐PET interpretation was limited by clinical premedication in a behaviorally constrained patient. For the initial FDG‐PET, documented premedication included amisulpride, diazepam, melatonin, clonidine, and intramuscular hydroxyzine, but no procedural sedation or motion artifact was documented. Current Society of Nuclear Medicine and Molecular Imaging (SNMMI)/European Association of Nuclear Medicine (EANM) guidelines recommend recording medication type and timing, particularly sedatives, psychotropic drugs, anti‐seizure medications, and corticosteroids, because medication effects on regional cerebral glucose metabolism have been suggested.^22^ Recent FDG‐PET cohort data also suggest that benzodiazepine exposure may be associated with altered brain metabolism.^23^ Medication effects therefore cannot be excluded, and the final FDG‐PET was limited by motion artifacts and reduced acquisition duration. Future multicenter studies combining clinical ratings, serial FDG‐PET, CSF biomarkers, EEG, and treatment timelines are needed.

## CONCLUSION

This case highlights the need to consider immune‐mediated etiologies in children with ASD presenting with atypical, severe, treatment‐resistant behavioral regression with catatonia‐like features. In antibody‐negative presentations, diagnosis should integrate clinical course, paraclinical findings, exclusion of alternatives, and multidisciplinary assessment. Serial FDG‐PET may support longitudinal monitoring, but remains nonspecific and treatment‐sensitive.

## PATIENT PERSPECTIVE

After clinical improvement, the patient discussed his hospitalization with his parents. According to them, he found admission emotionally difficult because of family separation and distress when visits ended. He also recalled infusion placement and the isolation room negatively, saying he felt unwell and understood he had to become calm before leaving. He nevertheless expressed trust in several staff members, described as “firm but kind,” and remembered walks and cafeteria snacks positively. He said he did not want to return to hospital, but added: “this hospital treated me.” He later reflected that he could better understand anger crises and should not hit others when upset.

## AUTHOR CONTRIBUTIONS

Edouard Guez contributed to the patient's clinical management, conceptualized the case report, reviewed the medical records, prepared the tables and figures, drafted the manuscript, and coordinated manuscript revisions. Theodore Falempin contributed to the patient's clinical management, provided clinical input on catatonic features, and critically revised the manuscript. Cyril Hanin contributed to the patient's clinical management before admission to the specialized neurodevelopmental crisis unit and critically revised the manuscript. Alison Arbouche, Rony Boucher, and Myriam Hamdi contributed to the patient's psychological, psychoeducational, and psychomotor assessment and care, contributed to the interpretation of behavioral and functional evolution, and critically revised the manuscript. Marie Raffin and Angèle Consoli contributed to the clinical interpretation of the case and critically revised the manuscript. Aurélie Kas contributed to the interpretation of nuclear medicine findings, including serial FDG‐PET imaging, prepared or validated the imaging figure, and critically revised the manuscript. Marianna Giannitelli supervised the patient's care in the specialized neurodevelopmental crisis unit, contributed to the therapeutic and clinical interpretation of the case, and critically revised the manuscript. David Cohen supervised the clinical and academic framing of the case report, contributed to the interpretation of the case, and critically revised the manuscript. All authors read and approved the final manuscript.

## CONFLICT OF INTEREST STATEMENT

The authors declare no conflicts of interest.

## ETHICS APPROVAL STATEMENT

Ethics approval was not required for this single‐patient case report. Written informed consent was obtained from the patient's legal guardians for publication of this case report and any accompanying anonymized clinical data and images.

## PATIENT CONSENT STATEMENT

Written informed consent was obtained from the patient's legal guardians for publication of this case report and any accompanying anonymized clinical data and images. A copy of the written consent is available for review by the Editor‐in‐Chief of this journal.

## CLINICAL TRIAL REGISTRATION

N/A.

## AI‐ASSISTED LANGUAGE EDITING

The authors used ChatGPT (OpenAI) to support language editing, readability, and manuscript organization. The authors reviewed and edited all content and take full responsibility for the final manuscript. No clinical data, medical interpretation, scientific conclusions, or submitted images were generated or modified by AI.

## Supporting information

Supporting File 1.

Supporting File 2.

## Data Availability

The clinical data used for this case report are not publicly available in order to protect patient confidentiality.
