# Supplementary material for: Serial FDG‐PET in probable seronegative autoimmune encephalitis presenting as severe behavioral regression in a child with autism
Source: PCN Rep. 2026 Sep 7;5(3):e70395. doi: 10.1002/pcn5.70395 (PMC13549223; doi:10.1002/pcn5.70395)
Supplement: Supplementary file 1 [file PCN5-5-e70395-s001.docx]

**Table S1. Chronology of treatments, symptoms, and paraclinical evolution**

| **Date/period** | **Treatment chronology and doses documented** | **Behavioral/psychiatric course** | **Neurological / imaging / work-up** |
| --- | --- | --- | --- |
| **Premorbid / baseline** | Baseline period before severe regression. Baseline supports: CMP follow-up since 2017; day-hospital care 2020-2023; human help in class, mainstream schooling with supports. | ASD diagnosed clinically in 2023 using DSM-5 clinical criteria. No baseline ADOS/ADI-R found in available material. No intellectual disability documented; good school level reported (reading, arithmetic, grammar). | History of febrile seizures in infancy; earlier EEG reportedly normal. |
| **Early September 2023 - mid July 2024** | Multiple psychotropics before transfer: aripiprazole, risperidone, sertraline, chlorpromazine, diazepam, haloperidol, topiramate, loxapine, olanzapine, fluoxetine, and valproate 750 mg/day introduced in mid/late May 2024. Exact start/stop dates incomplete for several agents. | Progressive severe behavioral/cognitive regression; aggression, self-injury, disorganization, eating from floor, refusal to dress, enuresis/encopresis, psychotic-like symptoms, refractory to several psychotropic strategies. | MRI in late April 2024 normal except small right frontal arachnoid cyst. Viral hepatitis in February 2024 with EBV/parvovirus B19 co-infection, rapid spontaneous improvement and liver normalization in ~15 days. |
| **Mid July 2024 - late September 2024** | Loxapine switched to levomepromazine; olanzapine adjusted 15 -> 10 -> 15 -> 10 mg; levomepromazine switched to chlorpromazine 30 mg three times daily; valproate/micropakote introduced in late July 2024 and increased to 400 mg twice daily by early August; clonidine introduced in early August; lithium LI 250 mg per tablet, one tablet morning introduced in mid September and increased in late September to 2 tablets morning + 1 tablet evening. | Severe agitation, self-injurious behavior and aggression toward others, compulsive loops, refusal of clothes, eating from floor, daily care impossible; multiple crisis protocols and restrictive/protective measures. | EEG in mid July 2024: normal background with bitemporal slow irregularities and occasional right frontocentral/bilateral sharp bursts, supporting focal epilepsy. EEG in late September 2024 after valproate: no epileptic abnormalities. |
| **Late September 2024 - late December 2024** | At FDG-PET in late October 2024, premedication: amisulpride 400 mg + diazepam 20 mg + melatonin 12 mg + clonidine 0.15 mg + hydroxyzine 50 mg IM. Other concomitant treatment doses were incompletely documented in the available records. | Persistent severe behavioral loops, aggression, hallucination-like phenomena, abnormal movements, refusal of care/medication; clinical picture refractory and fluctuating. | FDG-PET in late October 2024: moderate bilateral posterior associative cortical hypometabolism, more marked occipitally, and bilateral symmetric striatal hypermetabolism, described as compatible with a dysimmune process. The initial FDG-PET report did not document motion artifacts. Multidisciplinary clinical meeting in early November 2024: seronegative AE hypothesis and IVIG therapeutic trial. LP in late December 2024 traumatic/hemorrhagic; CSF biochemistry non-interpretable; PCR HSV1/HSV2/EBV negative; meningitis/encephalitis panel negative; antineuronal antibodies negative. |
| **Late January 2025** | IVIG course 1: Privigen 30 g/day for 4 days (total 120 g). Premedication: Polaramine. No adverse event reported. Lacosamide/Vimpat 200 mg/day added in late January because of concern for poor epilepsy coverage / unstable oral intake. Valproate LP 800 mg/day continued. | January-February remained highly symptomatic: severe loops, self-injurious behavior and aggression toward others. IM medication/restraint episodes, hallucination-like symptoms. | No immediate full response after first IVIG course documented. |
| **Mid February 2025** | IVIG course 2: Privigen 30-40-50 g over 3 days (total 120 g/cycle). Polaramine premedication. Hydration adjusted to shorten mechanical-restraint time during courses. | Still severe, but later weeks show partial behavioral improvement, especially afternoons; longer periods of communication possible. | No new EEG/PET data documented for this exact period. |
| **Mid March 2025 - late April 2025** | IVIG course 3: Privigen 30-40-50 g. Polaramine. After 3 courses: clinical improvement estimated at approximately 40%; symptom recrudescence in week before next course; plateau impression. | Mornings less dominated by crisis management; afternoons improved; fewer inappropriate behaviors; better medication intake and fewer injections. Persistent hallucination-like symptoms and loops; pre-infusion worsening before next IVIG. | EEG in late April 2025 reportedly normal, without focal or epileptic abnormality. |
| **Mid April 2025 - late May 2025** | IVIG course 4: mid April 2025, 30-40-50 g. IVIG course 5: mid May 2025, 30-40-50 g; olanzapine stopped for clozapine switch in late May 2025. | Continued improvement but plateau impression. Persistent fluctuations, loops, psychotic-like symptoms, and abnormal movements. | No new PET documented in April/May. Ongoing valproate/lacosamide. |
| **Late May 2025 - late June 2025** | Clozapine introduced in late May 2025; titrated progressively. IVIG course 6: mid June 2025, Privigen 30-40-50 g; hydration NaCl 500 ml/day noted. Amisulpride reportedly stopped progressively by late June 2025 after lack of efficacy. Diazepam 10 mg three times daily was used and tapered during 2025; exact taper dates not documented. | Substantial improvement but plateau. More accessible, less self-injurious behavior and fewer episodes of aggression toward others, fewer episodes requiring protective containment, fewer hallucination-like behaviors, and less disorganization; less intense and less frequent loops. | FDG-PET follow-up in late June 2025: marked improvement of posterior associative and striatal abnormalities; residual mild occipital associative hypometabolism, right-predominant; regression of striatal hypermetabolism. |
| **Mid July 2025 - late September 2025** | IVIG course 7: mid July 2025, 30-40-50 g. Course 8: mid August 2025, 30-40-50 g. Course 9: mid September 2025, 30-40-50 g. Clozapine titrated to 350 mg/day by late September 2025; clozapinemia within norms. Lithium decreased and stopped in early September 2025. Valproate decreased to 750 mg in mid September 2025. Clonidine reintroduced after August regression; dose not fully documented here. | Early clozapine period: sedation; first overnight leave in early July with no crisis at home except one self-injurious gesture. August transient regression: longer loops, abnormal movements, response latency, poorer availability, more soliloquy. September/October: persistent improvement, fewer and less intense loops, better transitions, rare psychotic features, outings/restaurant, permissions up to two nights. | Repeat CSF in late September 2025 showed mild pleocytosis, elevated CSF albumin compatible with blood-CSF barrier dysfunction, no clear intrathecal IgG synthesis, and negative neuronal autoantibodies. EEG in late September 2025: no epileptic abnormalities. |
| **Mid October 2025 - late November 2025** | IVIG course 10: mid October 2025. Rituximab first infusion in late October 2025: 750 mg/m2, capped at 1 g total; patient weight 67.5 kg, BSA 1.7562 m2, hence 1 g. Protocol: NaCl 1 L hydration from 08:00; Polaramine 5 mg, paracetamol 950 mg, methylprednisolone 65 mg 30 min pre-infusion; rituximab 1 g in 500 ml NaCl from ~09:00, escalating 25 -> 400 mg/h over ~4 h 45 min to 5 h. IVIG course 11 was documented in late November 2025; dosing details were not fully retrievable from the available records. | Around late October: increased irritability, majoration of loops, some return of hallucination-like behaviors, self-injurious behavior and aggression toward others, difficulty dressing, permissions reduced. Etiology retained: illness fluctuation and environmental/hospitalization burden rather than clear rituximab worsening; patient remained more available than earlier. | Rituximab well tolerated neurologically/hemodynamically; no severe infusion-related event. Multidisciplinary clinical meeting supported rituximab if AE considered possible/plausible, with 6-month reassessment using biomarkers including PET and MRI. |
| **Late December 2025 - late January 2026** | Home leave around holidays complicated by febrile viral syndrome. IVIG planned for early January 2026 was delayed because of a febrile viral syndrome and was administered in mid/late January 2026 according to the usual maintenance schedule. | Before delayed IVIG: increased loops, irritability, reduced social availability, frustration intolerance, hygiene/transition/treatment-related loops. Later schooling resumed in hospital in December 2025; regular weekend permissions, activities, restaurant visits reported. | FDG-PET in late January 2026: under limited conditions (motion artifacts and reduced acquisition duration) reported within normal limits overall; normal posterior associative cortex and striatal metabolism; mild nonspecific bilateral mediofrontal hypometabolism. |

**Note.** This supplementary table provides a chronological synthesis of documented treatments, behavioral/psychiatric evolution, and neurological, imaging, and laboratory findings. Dates and doses are reported as available from the medical records; when exact timing was unavailable, periods are approximate.
