# Supplementary material for: Serial FDG‐PET in probable seronegative autoimmune encephalitis presenting as severe behavioral regression in a child with autism
Source: PCN Rep. 2026 Sep 7;5(3):e70395. doi: 10.1002/pcn5.70395 (PMC13549223; doi:10.1002/pcn5.70395)
Supplement: Supplementary file 2 [file PCN5-5-e70395-s002.docx]

**Table S2. Etiological and diagnostic work-up**

| **Domain** | **Tests / assessments documented** | **Results available** |
| --- | --- | --- |
| **ASD diagnosis / neurodevelopmental baseline** | Clinical ASD diagnosis based on DSM-5 criteria. No baseline ADOS or ADI-R assessment was identified in the available records. CARS was available later during follow-up, with a total score of 24.5 in mid-July 2025. ABC irritability scores were 35 in late October 2024, 35 in late January 2025, 41 in late April 2025, 36 in late July 2025, and 27 in late October 2025. | No intellectual disability was documented. Before the current episode, the patient attended mainstream school with human support and had preserved academic abilities, including reading, arithmetic, and grammar. |
| **Clinical course supporting AE work-up** | Subacute/progressive rupture from baseline, severe regression, psychotic-like symptoms, abnormal movements, focal epilepsy, poor response to multiple psychotropics. | Multiple psychiatric treatment lines before immunotherapy without sustained improvement; deterioration with loss of adaptive skills and refractory self-injurious behavior and aggression toward others. |
| **MRI brain** | MRI in late April 2024. | No cerebral abnormality detected; small right superior paramedian frontal arachnoid cyst only; no ASL perfusion asymmetry; spectroscopy without abnormality. |
| **EEG / epilepsy** | EEG in mid July 2024; EEG in mid/late September 2024; EEG in late April 2025; EEG in mid/late September 2025. | Mid July: normal background with bitemporal slow irregularities and occasional right frontocentral/bilateral sharp bursts. Later EEGs after antiepileptic treatment: no epileptiform abnormalities documented. |
| **FDG-PET** | Late October 2024; late June 2025; late January 2026. | Initial PET: moderate bilateral posterior associative cortical hypometabolism, occipital-predominant, with bilateral symmetric striatal hypermetabolism. Follow-up: marked improvement of cortical and striatal abnormalities. Final: within normal limits overall despite motion artifacts/reduced acquisition duration; normal striatal metabolism; disappearance of posterior associative abnormalities; mild nonspecific bilateral mediofrontal hypometabolism. |
| **CSF - late December 2024** | LP under general anesthesia; traumatic/hemorrhagic sample. | Biochemistry was non-interpretable because of marked hemorrhagic contamination; leukocytes 50/mm³; no bacteria on direct examination; protein 18.6 g/L; glucose 3.6 mmol/L; lactate 2.28 mmol/L. Cytokines IL-1beta/IL-6/IL-10/TNF-alpha negative; interferon-alpha negative; PCR HSV1/HSV2/EBV negative; meningitis/encephalitis panel negative; Lyme negative; neuronal autoantibodies negative. Neurotransmitters not interpretable due to hemorrhagic sample; no neopterin increase. |
| **CSF - late September 2025** | Repeat CSF analysis with cell count, biochemistry, albumin/IgG indices, amino acids, neurotransmitters, pterins, and neuronal autoantibody testing. | Clear CSF with mild pleocytosis (8 leukocytes x10^6/L) and 0 red cells. Protein 0.36 g/L, glucose 3.4 mmol/L, CSF/serum glucose ratio 69%, lactate 1.6 mmol/L. CSF albumin was elevated at 169.4 mg/L; IgG 58.3 mg/L; IgG quotient 0.546; Reiber index interpreted as normal; transudation index 4.1707. Findings were considered compatible with elevated CSF albumin/blood-CSF barrier dysfunction, without clear intrathecal IgG synthesis. Neuronal autoantibodies remained negative. |
| **Blood / systemic infectious work-up** | February 2024 EBV/parvovirus B19 co-infection with hepatitis. Later blood/serology around late December 2024: HIV negative; CMV IgM negative/IgG positive; HSV1/2 IgG positive; VZV IgG positive; CMV PCR negative; blood EBV PCR positive at low level; EBV VCA/EBNA IgG positive; syphilis negative; Lyme negative; mycoplasma negative. Serum and CSF interferon-alpha testing negative. | Viral hepatitis was systemic/hepatic and resolved spontaneously with liver normalization in approximately 15 days. CSF PCR EBV was negative despite low-level blood EBV PCR positivity. No evidence supported active infectious encephalitis. |
| **Neuronal autoantibodies** | CSF: neuronal antibodies by indirect immunofluorescence, onconeuronal antibodies, anti-DPPX, anti-NMDA receptor, anti-AMPA1, anti-AMPA2, anti-CASPR2, anti-GABA, anti-LGI1, and autoimmune synaptopathy panel. Serum: neuronal antibodies by indirect immunofluorescence and immunodot, including anti-amphiphysin, anti-CV2, anti-Hu, anti-Ri, anti-Yo, anti-PNMA2, anti-SOX1, anti-GAD, anti-titin, anti-recoverin, anti-Tr/DNER, anti-Zic4, and anti-MOG antibodies. | All documented neuronal autoantibody tests were negative. |
| **Systemic autoimmune / NPSLE** | Antinuclear antibodies were tested by indirect immunofluorescence. Anti-dsDNA antibodies were measured by ELISA. ANCA were tested. | Antinuclear antibodies were detected at low titer by indirect immunofluorescence, with homogeneous and speckled patterns at 1:80. Anti-dsDNA antibodies were negative (4 IU/mL; negative threshold <20 IU/mL). ANCA were absent. No clinical or laboratory evidence supported neuropsychiatric systemic lupus erythematosus in the available records. |
| **Thyroid / Hashimoto encephalopathy exclusion** | TSH, free T4, free T3, anti-thyroid peroxidase antibodies, anti-thyroglobulin antibodies, and anti-TSH receptor antibodies. | Thyroid testing showed mildly elevated TSH with normal free T4 and free T3. Anti-thyroid peroxidase, anti-thyroglobulin, and anti-TSH receptor antibodies were negative. No thyroid antibody support for Hashimoto encephalopathy; no evidence of overt thyroid disease. |
| **Metabolic / nutritional work-up** | Copper normal; exchangeable copper low; REC 2.37%; vitamins A/B1/B6/E normal; B12 normal; folate low; lactate/pyruvate redox abnormal without increased L/P ratio; broad CSF amino acid, neurotransmitter, and pterin testing documented. | No clear metabolic etiology documented in available material; folate deficiency corrected. |
| **Genetic work-up** | Genetic work-up discussed: ACPA/exome/genome trio. Available result: UGT1A1 heterozygous variant consistent with Gilbert tendency, no implication in pathology. | No documented pathogenic genetic diagnosis in available material. |
| **Oncological / paraneoplastic screening** | Whole-body CT showed no tumor syndrome except an isolated right inguinal lymph node measuring 11 mm short axis / 19 mm long axis. AFP was mildly elevated on two measurements, 13.1 microg/L in late May 2025 and 14.4 microg/L in early September 2025, while LDH and hCG were normal. Targeted inguinal ultrasound showed a benign-appearing lymph node measuring 23 x 8 mm with preserved fatty hilum, homogeneous splenomegaly at 13 cm, and no mass or abdominopelvic effusion. Oncology advice was to repeat AFP monitoring at 3-6 months, without further immediate exploration. | No documented tumor syndrome or paraneoplastic source was identified in the available work-up. |
| **Hepatic / viral hepatitis follow-up** | Abdominal/hepatic ultrasound in mid October 2024: normal liver size/echostructure; no focal lesion; no biliary dilation; normal vessels; elastometry normal for age. Hepatology consultation in early April 2025: no argument for hepatitis C; HCV false-reactivity likely; clinical exam without liver disease; liver ultrasound and liver tests normal. | Supports resolved systemic infection/hepatitis and no ongoing hepatopathy. |

**Note.** This supplementary table summarizes the etiological and diagnostic work-up performed to assess possible immune-mediated, infectious, metabolic, genetic, systemic autoimmune, thyroid-related, and oncological/paraneoplastic causes. Results are reported according to the available medical records.

***NPSLE = neuropsychiatric systemic lupus erythematosus.***
